# Long noncoding RNA HOTTIP as a novel predictor of lymph node metastasis and survival in human cancer: a systematic review and meta-analysis

**DOI:** 10.18632/oncotarget.12981

**Published:** 2016-10-28

**Authors:** Zhicong Chen, Anbang He, Dailian Wang, Yuchen Liu, Weiren Huang

**Affiliations:** ^1^ Key Laboratory of Medical Reprogramming Technology, Shenzhen Second People's Hospital, The First Affiliated Hospital of Shenzhen University, Shenzhen, China

**Keywords:** lncRNA, HOTTIP, cancer, prognosis, lymph node metastasis

## Abstract

HOXA transcript at the distal tip (HOTTIP), a functional lncRNA transcribed from the 5′ tip of the HOXA locus, has been functionally characterized as an oncogene in various cancers. To further explore the clinical value of HOTTIP in cancer, we collected all relevant studies and investigated the association between HOTTIP level and lymph node metastasis (LNM) or overall survival (OS). Literature collection was conducted by searching electronic databases PubMed, Cochrane Library, OVID, Web of Science and Chinese National Knowledge Infrastructure (CNKI)(up to July 7, 2016). Seven studies with 652 cancer patients were included in the meta-analysis according to the inclusion and exclusion criteria. The results showed a significant positive association between HOTTIP levels and LNM (Odds ratio, OR = 2.30, 95 % CI: 1.58-3.35, *p* < 0.0001) in a fixed-effects model (I^2^ = 0 %, *p* = 0.949) and it could also predict poor OS in cancer patients (Hazard ratio HR = 2.24, 95% CI: 1.74–2.90, *p* < 0.00001) in a fixed-effects model (I^2^ = 0%, *p* = 0.925). In conclusion, this meta-analysis demonstrated that the higher expression level of HOTTIP is correlated with positive LNM and poor OS in different types of cancer and HOTTIP might serve as a novel predictor of LNM and survival in human cancer.

## INTRODUCTION

Nowadays, cancer is still a major public health problem, one leading cause of death all over the world, due to the increasing incidence and mortality. Estimated by GLOB-CAN 2012, there were 14.1 million new cancer cases, 8.2 million cancer deaths, and 32.6 million people living with cancer occurred in 2012 worldwide [1].It is predicted that there will be about 4.3 million newly diagnosed invasive cancer cases and 2.8 million cancer deaths in 2015 in China [2]. There is no doubt that the ideal cancer screening tools are definitely required in predictive, diagnostic and prognostic aspects. Among them, risk assessment and prognostication are indispensable for treatment decision making, patient counseling, and inclusion in clinical trials. Like TNM, a recognized cancer staging notation system, is widely used for adopting a global standard to give an indication of prognosis. However, common staging systems have been performed with limited prediction accuracy because it cannot incorporate novel information such as biomarkers or more complex bioinformatics. More and better prediction tools are needed to fulfill the accuracy and utility of prediction models for cancer.

A notable revolution has been sparked in the oncology field due to the identification and exploration of so many cancer-associated lncRNAs. Numerous evidences suggest that lncRNAs served as critical regulators in the development of different cancers by influencing diverse cellular processes, including cancer initiation and progression [3, 4]. HOXA transcript at the distal tip (HOTTIP), a lncRNA transcribed from the 5′ tip of the HOXA locus and serves as a key locus control element of HOXA genes and distal identity [5], has been recently functionally characterized by its tumor-relativity and involvement in carcinogenesis. Up-regulated expression of HOTTIP has been reported in different cancers, including hepatocellular carcinoma [6], pancreatic cancer [7], colorectal cancer [8, 9], osteosarcoma [10], tongue squamous cell carcinoma [11] and gastric cancer [12].All these data indicated that HOTTIP plays an potential oncogenic role in these tumors by promoting cell proliferation, inhibiting cell apoptosis and increasing cell migration. Together, lncRNA HOTTIP may not only act as a potential therapeutic target, but also as a novel prognostic biomarker in cancer. To shed light on these results and to more precisely evaluate the relationship between lncRNA HOTTIP and lymph node metastasis / survival in human cancer, we performed a meta-analysis of published studies.

## RESULTS

### Selection of studies

**Figure 1:**
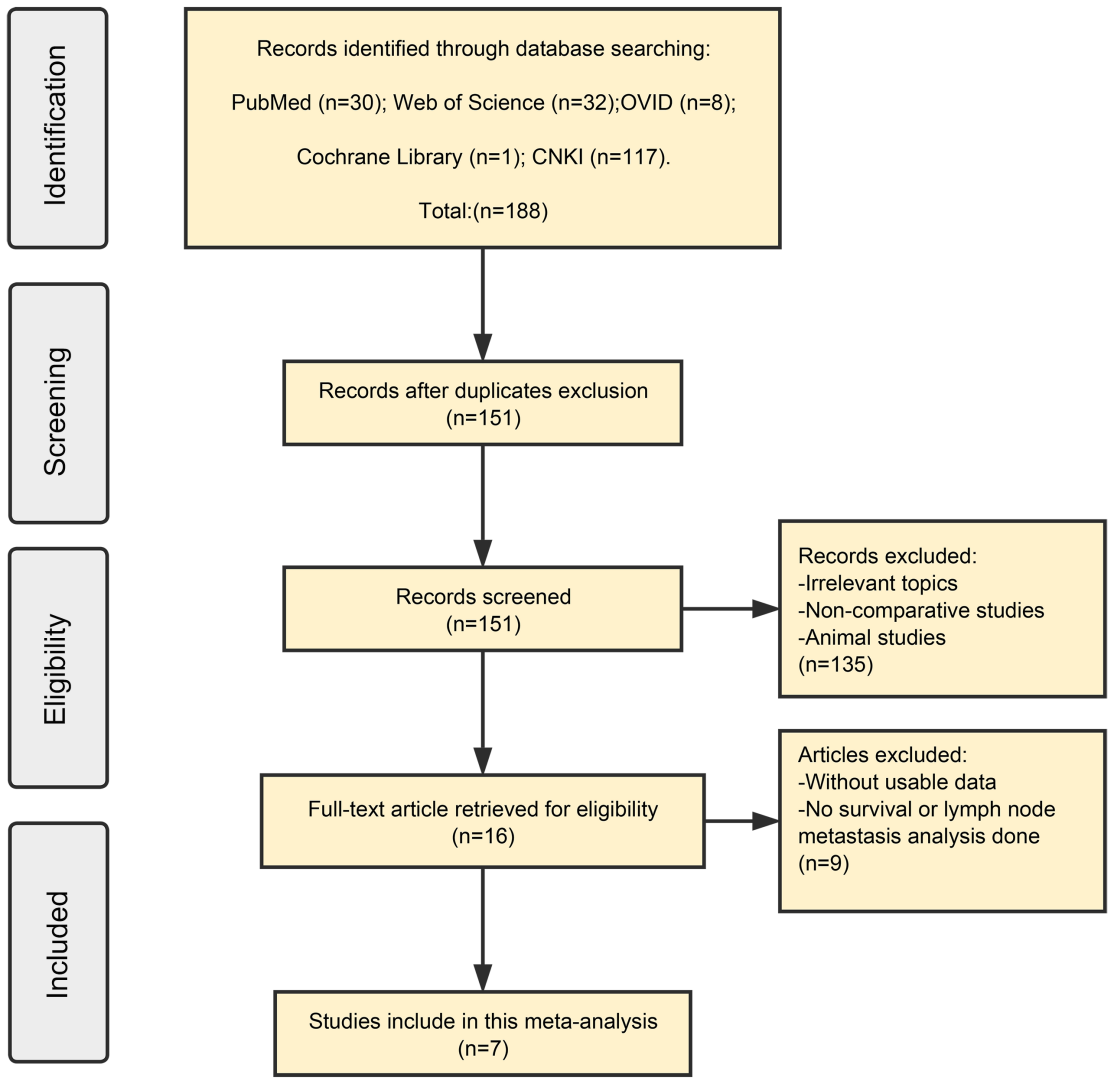
Flowchart of selecting studies for inclusion

A total of 188 records were retrieved from the below databases in initial search and 37 duplicate reports were excluded. After detailed screening of the title and abstract, irrelevant and non-comparative articles were excluded and 16 potential eligible studies were selected. After further evaluation of the full articles, a total of 7 publications addressing the relationship between lncRNA HOTTIP and cancer LNM or OS were found to meet all of the inclusion criteria and used for data extraction. All of the included studies were non-randomized. A flowchart of the study selection process is shown in Figure 1.

### Characteristics of eligible studies

All of the eligible studies were published recently (2014-2016). These studies included a total of 652 patients with a mean patient sample size of *N* = 93.14 (range 48 to 156). Six different types of cancer were evaluated in this meta-analysis: 2 colorectal cancers (CRC), 1 hepatocellular carcinoma (HCC), 1 pancreatic cancer(PC), 1 osteosarcoma(OSA), 1tongue squamous cell carcinoma(TSCC), and 1 gastric cancer (GC). All the detected samples were tissues or frozen tissues from the patients without anti-cancer treatments. The expression of HOTTIP was measured by RT-qPCR and normalized to GAPDH or β-action. Cut-off scores that discriminate between high and low HOTTIP were selected by ROC curve or median value or X-tile algorithm. Of the 7 included studies, not all studies examined both OS and LNM. All the diagnoses of lymph node metastasis were based on pathology. The main characteristics of the eligible studies were summarized in Table 1. The Newcastle-Ottawa Scale (NOS) confirmed that all the studies were of good quality Table 2.

**Table 1:** Characteristics of studies in this meta-analysis

Study	Year	Country	Cancer type	Total number	Detection method	Cut-off	HOTTIP expression				Survival analysis	Multivariate analysis	HR statistic	Hazard ratios (95% CI)	Follow-up, moths
							High expression	High with LNM	Low expression	Low with LNM					
Quagliata	2014	Switzerland	HCC	52	RT-qPCR	ROC curve	32	NA	20	NA	OS	No	Survival curve	1.560 (0.67-3.64)	80 (total)
Wang	2015	China	PC	144	RT-qPCR	ROC curve	118	75	26	10	OS	Yes	Data in paper	2.589 (1.385–4.839)	60 (total)
Ren	2015	China	CRC	156	RT-qPCR	Median	77	47	79	36	OS	Yes	Data in paper	2.151 (1.306-3.415)	46 (median)
Li	2015	China	OSA	68	RT-qPCR	Median	34	NA	34	NA	OS	Yes	Data in paper	2.887 (1.367-7.061)	60 (total)
Zhang	2015	China	TSCC	86	RT-qPCR	Median	44	28	42	18	OS	Yes	Data in paper	2.113 (1.062–3.115)	38 (median)
Lian	2016	China	CRC	48	RT-qPCR	X-tile algorithm	32	21	16	7	NA	NA	NA	NA	NA
Ye	2016	China	GC	98	RT-qPCR	Median	49	39	49	29	OS	Yes	Survival curve	2.420 (1.21-4.85)	60 (total)

HCC hepatocellular carcinoma, PC pancreatic cancer, CRC colorectal cancer, OSA osteosarcoma, TSCC tongue squamous cell carcinoma, GC gastric cancer, RT-qPCR real-time quantitative PCR, LNM lymph node metastasis, OS overall survival, NA not available.

**Table 2:** Methodological quality of the eligible studies according to the Newcastle-Ottawa scale

Author/year	Country	Adequacy of case definition	Representativeness of the cases	Selection of controls	Definition of controls	Comparability cases/controls	Ascertainment of exposure	Same method of ascertainment	Nonreponse rate
Quagliata	Switzerland	★	★	★	NA	★★	★	★	NA
Wang	China	★	★	★	NA	★★	★	★	NA
Ren	China	★	★	★	NA	★★	★	★	NA
Li	China	★	★	★	NA	★★	★	★	NA
Zhang	China	★	★	★	NA	★★	★	★	NA
Lian	China	★	★	★	NA	★★	★	★	NA
Ye	China	★	★	★	NA	★★	★	★	NA

Notes: This table identifies “high-quality” choices with a “star (*)”. A study can be awarded a maximum of one star for each numbered item within the selection and exposure categories. A maximum of two stars can be given for comparability. NA: not available

### Meta-analysis results

#### Association between lncRNA HOTTIP and LNM

**Figure 2:**
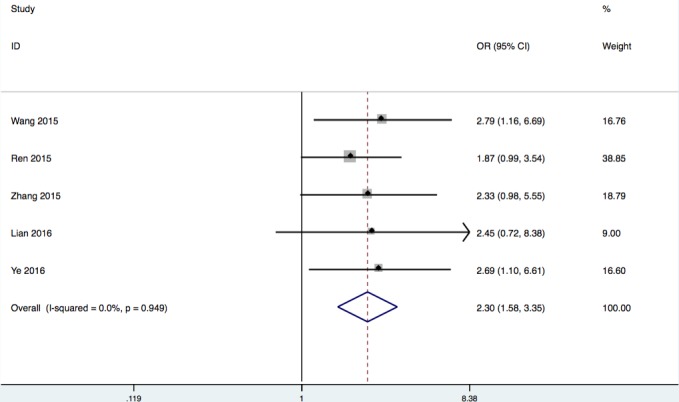
Forest plot of the correlation between HOTTIP expression levels and LNM in different cancer patients

Five studies reporting a total of 444 patients with LNM were included based on different HOTTIP expression patterns. The fixed-effects model was adopted as the nonsignificant heterogeneity (I^2^ = 0 %, *p* = 0.95). Analysis showed the OR of 2.30 with 95 % CI: 1.58-3.35 (*p* < 0.0001), which revealed that a higher HOTTIP expression was predictive of higher LNM (Figure 2). The result demonstrated that cancer patients with high HOTTIP expression in tumor tissues were more susceptibility to develop LNM.

#### Association between lncRNA HOTTIP and OS

**Figure 3:**
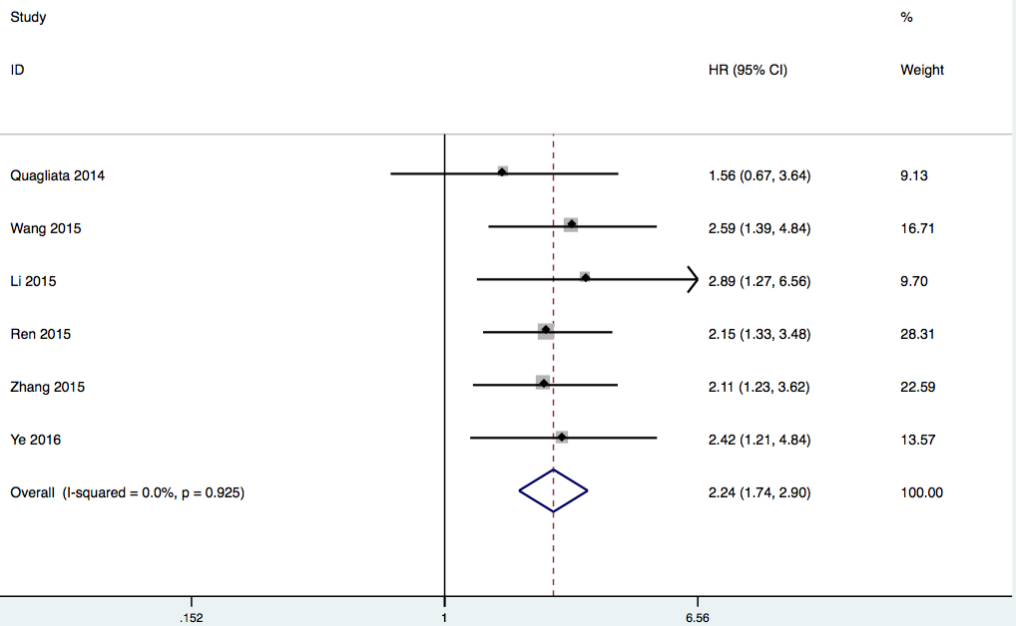
Forest plot of the correlation between HOTTIP expression levels and OS in different cancer patients

Six studies reporting a total of 604 patients with OS were included based on different HOTTIP expression levels. The relationship between HOTTIP expression and the OS of cancer patients was found to be of no significant heterogeneity (I^2^ = 0%, *P* = 0.92), and the fixed-effect model was therefore applied. Data of pooled HRs (HR = 2.24, 95% CI: 1.74-2.90, *P* < 0.00001) manifested that high expression of HOTTIP had a statistic shorter OS (Figure 3). In other words, high HOTTIP expression correlated with a worse survival.

#### Publication bias

**Figure 4:**
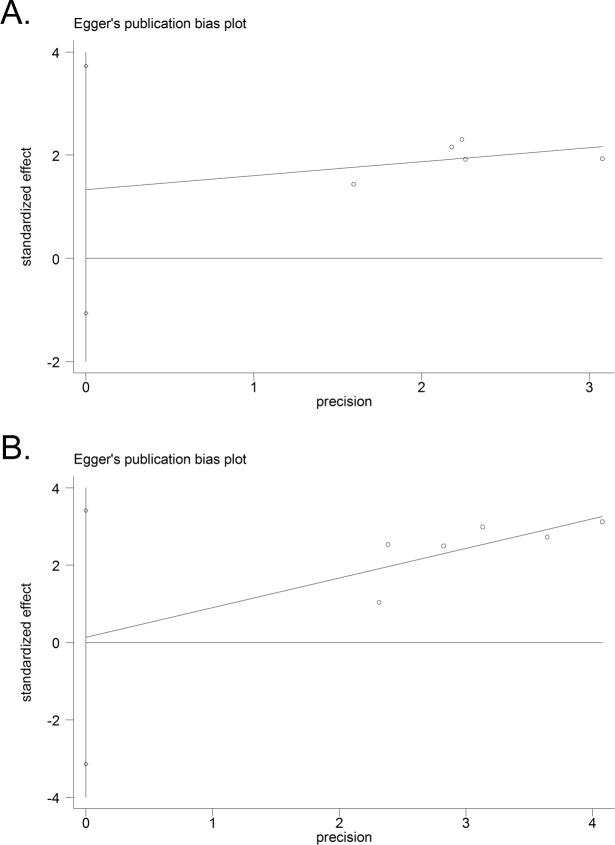
Funnel plot analysis of potential publication bias in LNM and OS group(Egger's test): A. LNM group; B. OS group

Egger's tests were performed to assess publication bias of the present meta-analysis. In both LNM group (Egger's test, *t* = 1.77, *p* = 0.176) and OS group (Egger's test, *t* = 0.11, *p* = 0.918), no significant publication bias was observed by the Egger's test (Figure 4).

## DISCUSSION

Since the completion of the ENCODE project, the noncoding RNAs have captured our great attention. LncRNAs, defined as non-coding transcripts surpass 200 nucleotides in length, have been indeed revealed in various aspects of cellular homeostasis in different cancers. Increasing tumor-associated lncRNAs have been functionally characterized and totally expanded cancer perspective. Meanwhile, accumulating evidences suggested that cancer-specific lncRNAs might serve as novel prognostic molecular markers [13–15]. Therefore, the comprehensive identification of cancer-associated lncRNAs may provide a groundbreaking method to more precisely evaluate the prognosis of different cancers.

HOTTIP is a well-known lncRNA which acts as a master regulator of HOXA genes. Recently, the involvement of the lncRNA HOTTIP in various cancers has been elucidated [6–12]. The further comprehensive mechanism between HOTTIP and cancer was reported in continuance. HoxA13-HOTTIP-IGFBP-3 cascade is critical for the carcinogenic characteristics in human gastric cancer [16]. HOTTIP plays a pivotal role in osteosarcoma cell initiation and chemoresistance via activating Wnt/β-catenin signaling pathway [17]. Further exploration of correlation between HOTTIP and cancer is definitely required.

In order to combine the results of previous studies about HOTTIP and cancer to arrive at a summary conclusion, we elucidated the relationships between HOTTIP expression levels and LNM and OS in cancer in the present meta-analysis. To the best of our knowledge, this is the first meta-analysis providing comprehensive insights into the correlation of lncRNA HOTTIP and cancer prognosis. The pooled data of eligible studies indicated that high HOTTIP expression was significant correlated with LNM (OR = 2.30, 95 % CI: 1.58-3.35, *p* < 0.0001), and OS (HR = 2.24, 95% CI: 1.74-2.90, *p* < 0.00001). High expression of HOTTIP predicted more prone to LNM and poor OS. Although HOTTIP was found to be significantly associated with the prognosis of cancer patients, some limitations in our meta-analysis should be mentioned. First and foremost, the eligible studies in this analysis were insufficient. Only seven studies and six types of cancer were included. Potential publication bias is likely to exist, in spite of no evidence obtained from our analysis. Due to inadequate data, we did not evaluate sensitivity bias. Additionally, most of the studies were conducted with Chinese sample populations and, consequently, our results may result in potential ethnic bias and only applicable in this ethnic group. What's more, the cut-off value of HOTTIP expression differed in these studies. Last but not least, many factors, such as treatment, and duration of follow- up, may also affect OS. Hence, the data of this meta-analysis should be updated and confirmed by following studies.

In conclusion, despite the limitations described above, our systematic review and meta-analysis reveals that LncRNA HOTTIP is significantly associated with LNM and OS in patients with diverse cancers, and could be used as a potentially and promising prognostic marker in human cancer. Nevertheless, large-volume, well-designed studies with extensive follow-up are awaited to confirm and update the findings of this analysis.

## MATERIALS AND METHODS

### Search strategy

A literature search was performed on PubMed, Cochrane Library, OVID, Web of Science and Chinese National Knowledge Infrastructure (CNKI) using the following search keyword: HOTTIP or HOXA transcript at the distal tips. The last update of searching time was July 7, 2016.

### Inclusion and exclusion criteria

Inclusion criteria are as the following: (1) Article investigating the association between HOTTIP expression and prognosis of patients cancer; (2) Cancer patients were grouped according to the expression levels of HOTTIP which were measured in primary tumor tissues; (3) Related clinical parameters were described, including LNM or OS; (5) Sufficient data were contained for the computation of OR, HR and corresponding 95 % CI. Exclusion criteria are as the following: (1) Duplicate publications; (2) Letters, editorials, expert opinions, case reports and reviews; (3) Irrelevant or non-comparative or nonhuman research; (4) Studies without usable data.

### Date extraction

Data were extracted independently by three authors (CZC, HAB, and WDL), according to the inclusion and exclusion criteria. Disagreements were resolved by two investigators (LYC,HWR) by discussions. The following data were extracted: (1) Publication information: first author's last name, year of publication, country; (2) Patients’ characteristics: cancer type; number of participants, detected sample and follow-up duration; (3) HOTTIP expression measurement and cut-off value; (4) ORs of HOTTIP for LNM: number of patients with LNM in each group; (5) HRs of HOTTIP for OS as well as their 95% CIs and P values. If only Kaplan-Meier curves were available, we extracted data from the graphical survival plots and estimated the HRs. All the calculations mentioned above were based on the methods illustrated by Parmar et al. [18] and Tierney et al. [19]

### Statistical methods

A test of heterogeneity of combined ORs or HRs was conducted using Cochran's Q test and Higgins I-squared statistic. *P* values < 0.1 was considered significant. I^2^ values >50% indicate heterogeneity among studies. A fixed effect model was applied when heterogeneity might not be important between studies (*P* > 0.1), while the random effect model was used if heterogeneity was observed (*P* < 0.1). The potential publication bias was assessed by the Egger's test. All the statistical analyses were carried out by using the Stata 12.0. *P* values < 0.05 were considered statistically significant.
